# Bilateral decompressive craniectomy in pediatric patients: A systematic review

**DOI:** 10.1007/s10143-026-04335-5

**Published:** 2026-05-30

**Authors:** Yaxel Levin-Carrion, Kevin Titkov, Isabella Chamma, Ashutosh Gajbinker, Jayant Bhasin, Arman Sawhney, Daniel J. Valdivia, Gabriela Carrillo, Daniela Alejandra Perez-Chadid, Drew Thibault, Alejandro Pando, Nemanja Novakovic, Hai Sun

**Affiliations:** 1 Department of Neurological Surgery, Rutgers New Jersey Medical School, Newark, NJ USA; 2 Department of Neurological Surgery, Johnson Medical School, Rutgers Robert Wood, New Brunswick, NJ USA; 3 Department of Internal Medicine, Rutgers New Jersey Medical School, Newark, NJ USA; 4 Department of Neurological Surgery, Corewell Health William Beaumont University Hospital, , Royal Oak, MI USA

**Keywords:** Pediatric Decompressive Craniectomy, Bifrontal Craniectomy, Bilateral Craniectomy, Traumatic Brain Injury (TBI), Functional outcomes, Pediatric decompression

## Abstract

Traumatic brain injury (TBI) and other acute neurological insults remain leading causes of pediatric morbidity and mortality. In cases where elevated intracranial pressure (ICP) is refractory to maximal medical therapy, decompressive craniectomy (DC) may be considered as a salvage intervention. Although bilateral, bifrontal, bitemporal, and cruciate decompressive approaches are used in selected patients with diffuse cerebral edema or bihemispheric swelling, their role, timing, complications, and outcomes remain poorly defined in pediatric populations. Following PRISMA guidelines, PubMed, Scopus, Embase, and PubMed Central were searched for English-language studies reporting bifrontal, bitemporal, bilateral, or cruciate decompressive craniectomy in pediatric or young adult patients, defined a priori as age ≤ 21 years, with refractory intracranial hypertension from traumatic or non-traumatic etiologies. Studies were included only if the cohort met this age criterion or if pediatric/young adult outcomes could be separately extracted. Mixed-age studies without extractable pediatric/young adult data were excluded from the primary synthesis. Data regarding demographics, operative technique, timing, mortality, complications, ICP response, and functional outcomes were extracted and synthesized qualitatively because of clinical and methodological heterogeneity. Five studies met eligibility criteria for the primary pediatric/young adult synthesis, including one small randomized pilot trial and four observational cohorts. The available evidence was heterogeneous with respect to indication, ICP threshold, timing of surgery, operative technique, and outcome measurement. Bitemporal, bifrontal, bilateral, and cruciate decompressive strategies were reported primarily in selected patients with severe traumatic brain injury or diffuse cerebral swelling. Several larger pediatric decompressive craniectomy cohorts included only small bilateral or bifrontal subgroups, and outcomes were not always stratified by technique. Reported mortality, functional recovery, hydrocephalus, subgaleal collections, and need for reoperation varied across studies. Early decompression was associated with ICP reduction and favorable outcomes in limited reports, including one small randomized trial, but the evidence remains insufficient to determine comparative efficacy, optimal timing, or superiority of one bilateral decompressive approach. Bilateral, bifrontal, bitemporal, and cruciate decompressive craniectomy have been reported as feasible salvage strategies for carefully selected pediatric and young adult patients with refractory intracranial hypertension. However, current evidence is sparse, heterogeneous, and often not technique-specific. Therefore, definitive conclusions regarding efficacy, optimal timing, or superiority of one bilateral approach cannot be made. Prospective multicenter studies with standardized age definitions, ICP thresholds, operative taxonomy, complication reporting, and long-term neurocognitive outcomes are needed.

**Supplementary Information:**

The online version contains supplementary material available at 10.1007/s10143-026-04335-5.

## Introduction

Intracranial hypertension secondary to cerebral edema is a frequent and feared complication of severe head injury and other acute brain disorders in patients of all age groups [1]. Cerebral edema is commonly seen on imaging following traumatic brain injury and may contribute to uncontrolled ICP elevation [2, 3]. Sustained intracranial hypertension can impair cerebral perfusion, worsen secondary ischemic injury, and ultimately lead to brain herniation and death [4]. Children may be particularly vulnerable to acute intracranial hypertension because they often lack the degree of cerebral atrophy that can provide compensatory intracranial reserve in older adults [5–7]. First-line management of elevated ICP is similar in adults and children and includes optimization of ventilation, osmotic therapy, sedation, cerebrospinal fluid diversion when appropriate, and treatment of systemic factors that may worsen cerebral edema [8, 9]. In some patients, however, maximal medical therapy fails to adequately control ICP, prompting consideration of decompressive craniectomy.

In adults with severe TBI, large decompressive procedures have been studied in randomized trials [10, 11]. These studies have produced nuanced findings, including reductions in ICP and mortality in some contexts, but also concern for survival with severe disability [10, 11]. As a result, the role of decompressive craniectomy remains complex and highly dependent on patient selection, timing, injury pattern, and goals of care [12, 13]. Adult data cannot be directly extrapolated to children because pediatric patients differ in injury mechanisms, skull and brain development, cerebral compliance, recovery potential, and long-term neurocognitive considerations [14].

The pediatric literature is more limited. Existing reports include small randomized trials, retrospective cohorts, and single-center institutional experiences. Furthermore, the term bilateral decompressive craniectomy encompasses several non-identical operative approaches, including bifrontal decompression, bitemporal decompression, bilateral hemicraniectomy, bilateral frontotemporoparietal decompression, and bifrontal-biparietal cruciate decompression. These techniques may be used for different radiographic patterns, including diffuse cerebral edema, bilateral swelling, midline mass effect, refractory intracranial hypertension, or selected traumatic lesions.

The purpose of this systematic review is to synthesize the available literature on bilateral, bifrontal, bitemporal, and cruciate decompressive craniectomy in pediatric and young adult patients. Given the limited and heterogeneous nature of the evidence, this review focuses on feasibility, reported outcomes, complications, and limitations of the current literature rather than attempting to establish definitive efficacy or superiority of any operative approach.

## Methods

**Fig. 1:**
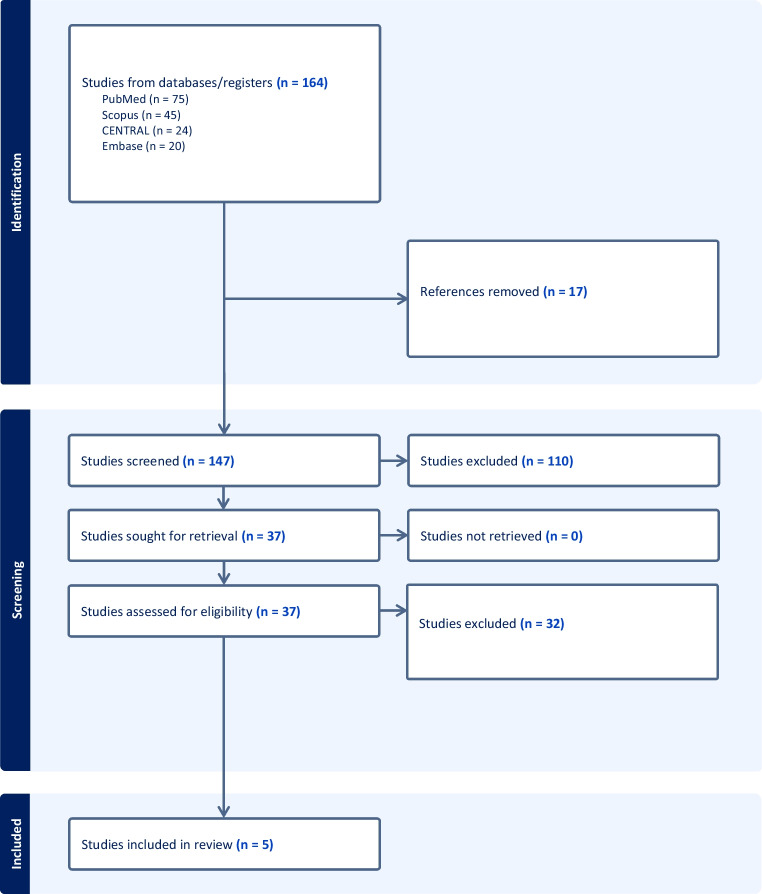
PRISMA Flow Diagram of Included Studies

This systematic review was conducted in accordance with PRISMA principles. PubMed, PubMed Central, Scopus, and Embase were searched for English-language studies published through September 9, 2025. Search terms combined concepts related to decompressive craniectomy, bifrontal or bilateral operative approaches, traumatic brain injury or intracranial hypertension, pediatric or adolescent populations, and clinical outcomes. The full search syntax is provided in the Supplementary Table 1. Search results were imported into Zotero and deduplicated. The initial search identified 164 records, including 75 from PubMed, 45 from Scopus, 24 from PubMed Central, and 20 from Embase. After removal of 17 duplicates, 147 titles and abstracts were screened. One hundred nine studies were excluded during title and abstract screening, and 38 articles underwent full-text review. Ten reports were initially identified as potentially relevant to bilateral, bifrontal, bitemporal, or cruciate decompressive craniectomy. After applying age and extractability criteria, five studies were included in the primary pediatric/young adult synthesis (Fig. 1).

### Eligibility criteria

The population of interest was defined a priori as pediatric or young adult patients aged ≤ 21 years. This age threshold was selected because several pediatric neurosurgical series include older adolescents and young adults through age 21. Studies were eligible if they reported outcomes after bifrontal, bitemporal, bilateral hemicraniectomy, bilateral frontotemporoparietal, or cruciate decompressive craniectomy for refractory intracranial hypertension. Studies were included in the primary synthesis only if the entire cohort met the age criterion or if pediatric/young adult outcomes could be separately extracted. Mixed-age cohorts without extractable pediatric/young adult data were excluded from the primary synthesis. Case reports, animal studies, non-English studies, studies without extractable outcome data, and studies limited to unilateral decompressive craniectomy without relevant bilateral, bifrontal, bitemporal, or cruciate data were excluded. Studies that included adult or mixed-age cohorts without separately extractable pediatric/young adult outcomes were not used to support pediatric outcome conclusions. When such studies were relevant to operative technique or historical background, they were listed separately as contextual literature and excluded from the primary analysis.

### Data extraction

Two independent reviewers screened eligible articles and extracted study characteristics, including study design, country or region, population, age range, indication for surgery, operative technique, timing of decompression, ICP metrics, mortality, functional outcomes, return to school when reported, complications, and predictors of poor outcome. Discrepancies were resolved by consensus.

### Synthesis and risk of bias

Because operative indications, ICP thresholds, surgical techniques, timing, and outcome metrics varied substantially, quantitative meta-analysis was not performed. Instead, studies were synthesized narratively, with particular attention to whether outcomes were specific to bilateral, bifrontal, bitemporal, or cruciate decompression or reflected broader pediatric decompressive craniectomy cohorts. The randomized pediatric pilot trial was considered separately from observational studies because it provided the only controlled pediatric evidence, though its small sample size limited generalizability. The observational studies, which we rated with an adapted Newcastle–Ottawa/ROBINS-I approach, were interpreted cautiously because of retrospective design, confounding by indication, inconsistent ICP monitoring protocols, variable thresholds for surgery, heterogeneous postoperative management, and incomplete long-term follow-up. The overall certainty of evidence was judged to be low because of small sample sizes, observational design in most reports, heterogeneity in operative technique and indication, and limited technique-specific outcome reporting. Therefore, causal conclusions regarding efficacy, optimal timing, or superiority of any bilateral decompressive technique were not considered appropriate.

## Results

### Study characteristics

After applying the age and extractability criteria, five studies were included in the primary pediatric/young adult synthesis. These included one small randomized pilot trial and four observational cohorts. The studies varied substantially in design, indication, surgical technique, and outcome reporting. Detailed study-level characteristics, including study design, sample size, age range, operative approach, indication, and whether bilateral/bifrontal outcomes were stratified, are provided in Table 1.

**Table 1:** Primary Pediatric/Young Adult Studies Included in the Evidence Synthesis

Study	Design	Sample Size	Age	Operative approach	Indication	Bilateral/Bifrontal outcomes stratified?	Region
Taylor 2001	Randomized pilot trial	27	Median 120.9 months; range 13.6–176.4 months	Bitemporal decompressive craniectomy	Pediatric TBI with sustained intracranial hypertension	Yes; randomized surgical arm	Australia
McHugh 2019	Observational cohort	18	Median 14 years; range 0.25–21 years	Bifrontal-biparietal cruciate decompressive craniectomy	Severe TBI with diffuse edema, herniation, hemorrhage, or skull fracture	Yes	USA
Korhonen 2022	Cohort	24	Median 16 years; range 7.5–17.7 years	6 bifrontal one-flap, 5 bifrontal two-flap, 1 bilateral hemicraniectomy; other procedures also included	Refractory intracranial hypertension, predominantly severe TBI	Partially; cohort includes non-bilateral approaches	Finland
Konar 2024	Cohort	128	Mean 10.5 years in trauma group; 11.4 years in non-trauma group	7 bifrontal decompressive craniectomies; remainder largely unilateral	Pediatric trauma and non-trauma causes of intracranial hypertension	No; bifrontal subgroup not separately reported	India
Hubertus 2022	Cohort	48 total; 11 underwent DC	Median 4 years overall, range 0–16; DC subgroup median 5 years, range 0–13	9 hemicraniectomy; 2 bifrontal decompressive craniectomy	Severe pediatric TBI	No; only 2 bifrontal cases	Germany

Taylor et al. evaluated early bitemporal decompressive craniectomy in children with TBI and sustained intracranial hypertension [15]. McHugh et al. described a bifrontal-biparietal cruciate decompressive technique in pediatric and young adult patients with severe TBI [16]. Korhonen et al. reported outcomes after decompressive craniectomy in a predominantly adolescent pediatric cohort, including bifrontal one-flap, bifrontal two-flap, and bilateral approaches [19]. Konar et al. described a large pediatric decompressive craniectomy cohort that included a small bifrontal subgroup [20]. Hubertus et al. examined decompressive craniectomy in children with severe TBI, including a small number of bifrontal procedures [22]. Five studies initially identified as relevant were removed from the primary pediatric/young adult synthesis because they were mixed-age or predominantly adult cohorts without separately extractable pediatric/young adult outcomes. These included Timofeev et al., Ammar et al., Grille et al., Sarma et al., and Rubiano et al. [17, 18, 21, 23, 24] These studies are listed separately in Supplementary Table 2 and were not used to support pediatric efficacy conclusions.

### Surgical techniques and timing

Across included studies, bilateral or bifrontal decompression was not a uniform intervention. Reported approaches included bitemporal decompression, bifrontal one-flap or two-flap decompression, bilateral hemicraniectomy, bilateral frontotemporoparietal decompression, and bifrontal-biparietal cruciate decompression. Because no included study directly compared these techniques in a controlled fashion, the available data do not support conclusions regarding superiority of one operative approach over another.

Taylor et al. performed bitemporal decompressive craniectomy as an early intervention for children with TBI and sustained intracranial hypertension [15]. McHugh et al. described a bifrontal-biparietal cruciate technique designed to provide global decompression while preserving bone struts [16]. Korhonen et al. reported that a subset of patients underwent bifrontal one-flap or bifrontal two-flap decompression, while other patients underwent different decompressive procedures tailored to lesion location or swelling pattern [19]. Konar et al. reported only seven bifrontal procedures among 128 pediatric decompressive craniectomy cases, emphasizing the infrequent use of bifrontal decompression even in large pediatric series [20]. Hubertus et al. reported two bifrontal cases among 11 children who underwent decompressive craniectomy [22].

Timing of surgery varied across studies. Taylor et al. evaluated very early decompression, with surgery performed at a median of approximately 19 h after injury [15]. Korhonen et al. reported surgery at a median of 1.6 days, with approximately one-quarter of cases performed within 24 h [19]. Other studies did not provide sufficient bilateral-technique-specific timing data to support comparative conclusions. Overall, the literature suggests that early decompression may reduce ICP in selected patients, but current evidence does not establish that early bilateral decompression independently improves survival or functional outcome.

### Mortality

Mortality varied across studies and was not always reported specifically for bilateral or bifrontal subgroups. In Korhonen et al., three of 24 patients died, corresponding to an overall mortality of 12.5% in the pediatric decompressive craniectomy cohort [19]. In McHugh et al., three of 18 patients died after bifrontal-biparietal cruciate decompressive craniectomy [16]. Konar et al. reported mortality of 25.8% among trauma patients and 22.6% among non-trauma patients in the overall pediatric decompressive craniectomy cohort, but outcomes were not stratified by bifrontal technique [20]. Hubertus et al. reported in-hospital mortality of 27% among children who underwent decompressive craniectomy, compared with 11% among those who did not undergo decompression, though this comparison is limited by small sample size and confounding by indication [22]. Taylor et al. did not primarily report mortality as the main endpoint but found that none of the children in the decompressive craniectomy group remained vegetative at follow-up [15]. Because the trial was small, its findings should be interpreted as controlled but preliminary evidence rather than sufficient evidence to establish an independent mortality or functional outcome effect.

### Functional outcomes

Functional outcomes were assessed using variable measures, most commonly the Glasgow Outcome Scale, modified Glasgow Outcome Scale, or Glasgow Outcome Scale-Extended. In Korhonen et al., 15 of 24 children achieved good recovery, defined as Glasgow Outcome Scale-Extended score ≥ 7, and 86% of survivors returned to school [19]. However, these outcomes reflected the overall pediatric decompressive craniectomy cohort and were not exclusively bilateral-specific. Taylor et al. reported that seven of 13 children in the decompressive craniectomy group were normal or had mild disability at six months, compared with two of 14 children in the medical therapy group [15]. This trial also reported greater ICP reduction in the decompressive craniectomy group over the first 48 h [15]. Although this represents the only controlled pediatric evidence, the small sample size limits the strength and generalizability of efficacy conclusions.

Konar et al. reported favorable outcomes in 53% of trauma patients and 46.8% of non-trauma patients in the overall pediatric decompressive craniectomy cohort [20]. However, only seven patients underwent bifrontal decompression, and outcomes were not stratified by operative technique. Hubertus et al. reported no significant long-term Glasgow Outcome Scale difference between children who underwent decompressive craniectomy and those who did not, though the study included only a small number of decompressed patients and only two bifrontal cases [22]. McHugh et al. reported that 15 of 18 patients survived after cruciate decompression and 14 were discharged to rehabilitation, but long-term functional outcomes were not quantified in the same manner as in other studies [16].

Overall, the available literature documents survival and functional recovery in selected pediatric and young adult patients after bilateral, bifrontal, bitemporal, or cruciate decompression. However, because outcomes are inconsistently stratified by technique and most studies are observational, the evidence does not allow firm conclusions regarding the independent effect of bilateral decompression on long-term functional outcome. Study-level mortality, favorable outcome rates, and whether outcomes were bilateral-technique specific are summarized in Table 2.

**Table 2:** Outcomes Reported in Pediatric/Young Adults

Study	Bilateral/bifrontal/cruciate group	Mortality	Favorable Outcome	Interpretation
Taylor 2001	13 surgical patients underwent bitemporal DC	Mortality not primary endpoint; no vegetative survivors in DC group	7/13 (54%) normal or mild disability vs 2/14 (14%) in medical arm	Only controlled pediatric evidence; small sample limits generalizability
McHugh 2019	18 cruciate DC patients	3/18 (16.7%)	15/18 survived; 14 discharged to rehabilitation	Technique-specific but limited long-term functional data
Korhonen 2022	11 bifrontal patients plus 1 bilateral hemicraniectomy within broader cohort	3/24 overall (12.5%)	15/24 (63%) good recovery; 86% of survivors returned to school	Favorable cohort outcomes, but not fully technique-specific
Konar 2024	7 bifrontal DC patients among 128 children	25.8% trauma mortality; 22.6% non-trauma mortality	53% favorable outcome in trauma group; 46.8% in non-trauma group	Outcomes are cohort-level, not bifrontal-specific
Hubertus 2022	2 bifrontal cases among 11 DC patients	27% in-hospital mortality in DC group	Favorable outcome approximately 57% among DC patients at 12–36 months	Small DC subgroup; bifrontal outcomes not separately reported

### Complications

Complications were common but variably reported. Korhonen et al. documented complications in 11 of 21 survivors, with subcutaneous or subgaleal cerebrospinal fluid collections being among the most frequent [19]. Hydrocephalus requiring shunt placement occurred in a subset of patients and was associated with poorer functional recovery and failure to return to school [19]. Konar et al. reported complications in approximately 21% of trauma patients and 26% of non-trauma patients [20]. Reported complications included wound infection, hydrocephalus, bone flap osteomyelitis, and pseudomeningocele. However, these complications were reported for the overall pediatric decompressive craniectomy cohort and were not specific to the small bifrontal subgroup [20]. Hubertus et al. reported hydrocephalus more frequently among children who underwent decompressive craniectomy than among those who did not, though the small sample size limits interpretation [22]. McHugh et al. did not identify major surgical complication patterns in detail, but reported one infection and significant postoperative ICP reduction after cruciate decompression [16]. Taylor et al. did not systematically report surgical complications in the same level of detail as later observational studies [15].

Taken together, the available data suggest that hydrocephalus, subgaleal or subcutaneous fluid collections, infection, pseudomeningocele, and need for additional procedures should be anticipated and carefully monitored after decompressive craniectomy in children. The literature does not allow reliable comparison of complication rates between bifrontal, bitemporal, bilateral hemicraniectomy, and cruciate techniques. Major reported complications and adverse prognostic factors across the included pediatric/young adult studies are summarized in Table 3.

**Table 3:** Major Complications and Predictors in Included Studies

Study	Major complications reported	Key predictors or adverse factors
Taylor 2001	Surgical complications not systematically reported; greater ICP reduction reported after DC	Early decompression associated with ICP reduction, but sample size limits causal conclusions
McHugh 2019	No major complication pattern reported; one infection noted	Mortality and ICP trends described; no regression analysis of predictors
Korhonen 2022	Complications in 52% of survivors; subcutaneous/subgaleal CSF collections common; hydrocephalus requiring shunt in a subset	Shunt-requiring hydrocephalus associated with poor recovery and failure to return to school
Konar 2024	Complications in approximately 21–26%; wound infection, hydrocephalus, bone flap osteomyelitis, pseudomeningocele	GCS < 8 and motor score < 3 associated with poor outcome in trauma; motor score < 3 associated with poor outcome in non-trauma; pupillary asymmetry and hypotension associated on univariate analysis
Hubertus 2022	Hydrocephalus more frequent after DC than without DC	Small sample size limited predictor analysis

### Predictors of outcome

Several studies identified factors associated with unfavorable outcome, though most predictors were not specific to bilateral decompressive techniques. In Konar et al., Glasgow Coma Scale score < 8 and motor score < 3 were associated with unfavorable outcome in trauma patients, while motor score < 3 was associated with unfavorable outcome in non-trauma patients [20]. Pupillary asymmetry and hypotension were also associated with poorer outcomes in univariate analyses [20]. Korhonen et al. found that shunt-requiring hydrocephalus was associated with poorer recovery and failure to return to school [19]. The same cohort also suggested that radiographic severity, including compressed or absent basal cisterns, may be relevant to prognosis [19]. Hubertus et al. did not identify bilateral-specific predictors because of the small number of bifrontal cases [22]. McHugh et al. described postoperative ICP reduction after cruciate decompression but did not provide a regression analysis of predictors [16]. Overall, low presenting neurological status, impaired motor response, hypotension, pupillary abnormalities, persistent or postoperative intracranial hypertension, and hydrocephalus appear repeatedly as adverse factors across pediatric decompressive craniectomy literature. However, the current evidence does not determine whether these predictors should alter the decision to perform bilateral rather than unilateral decompression.

## Discussion

This systematic review summarizes the available literature on bilateral, bifrontal, bitemporal, and cruciate decompressive craniectomy in pediatric and young adult patients with refractory intracranial hypertension. The principal finding is that these procedures have been reported as feasible salvage strategies in selected patients, most commonly in the setting of severe TBI or diffuse cerebral swelling. However, the evidence base remains limited and heterogeneous. Most studies are observational, several include mixed decompressive techniques, and technique-specific bilateral outcomes are often unavailable. Therefore, this review should not be interpreted as demonstrating definitive efficacy.

The strongest pediatric evidence comes from the small randomized pilot trial by Taylor et al., which compared early decompressive craniectomy with continued medical management in children with TBI and sustained intracranial hypertension [15]. The trial reported greater ICP reduction and a higher proportion of normal or mildly disabled survivors in the surgical arm [15]. These findings are clinically important but must be interpreted cautiously because of the small sample size and limited generalizability. The remaining studies provide observational evidence that selected pediatric patients may survive and recover after bilateral or bifrontal decompressive procedures, but they cannot establish causality.

Timing remains unresolved. Early decompression was associated with ICP reduction in limited reports, particu­larly in Taylor et al. [15]. Korhonen et al. also reported favorable long-term recovery in a predominantly adolescent cohort in which some patients underwent early decompression [19]. However, selection bias, differences in injury severity, and center-specific protocols limit conclusions regarding whether timing independently improves survival or functional outcome. These data raise the possibility that earlier decompression may improve ICP control in selected patients, but they do not establish that early bilateral decompression independently improves long-term recovery.

Operative approach also remains heterogeneous. Bitemporal decompression, bifrontal one-flap or two-flap decompression, bilateral hemicraniectomy, bilateral frontotemporoparietal decompression, and bifrontal-biparietal cruciate decompression may differ in decompressive volume, temporal extension, venous sinus exposure, bone preservation, and cranioplasty implications. Yet the current literature does not directly compare these approaches. For diffuse edema or midline swelling refractory to medical management and without a discrete evacuable mass lesion, bifrontal or bihemispheric decompression may be considered as an individualized rescue strategy; however, current pediatric data do not establish superiority over continued medical therapy or alternative decompressive approaches.

Patient selection should be individualized and multidisciplinary. Factors such as presenting Glasgow Coma Scale score, motor response, pupillary examination, ICP trajectory, radiographic swelling pattern, basal cistern effacement, presence of mass lesions, systemic hypotension, coagulopathy, and family goals of care are all relevant. The available pediatric literature suggests that low presenting neurological status, hypotension, hydrocephalus, and persistent postoperative intracranial hypertension are associated with poorer outcomes [19, 20, 22]. However, these factors should not be interpreted as absolute contraindications without considering the broader clinical context.

Complications also remain an important consideration. Hydrocephalus, subgaleal or subcutaneous cerebrospinal fluid collections, wound infection, pseudomeningocele, bone flap complications, and need for reoperation were reported across studies [19, 20, 22]. Some complications were managed conservatively, whereas others required shunting or additional surgery. Because pediatric survivors may live for decades after injury, complication surveillance and long-term follow-up are particularly important. Technical variables may influence decompression adequacy, postoperative ICP control, and complication profiles, but existing pediatric data do not permit firm conclusions about their independent effect on outcomes. Future studies should therefore report flap dimensions, temporal extension, duraplasty technique, bone-sparing modifications, timing of cranioplasty, and whether mass lesions were evacuated at the time of decompression. Standardized operative taxonomy would improve comparability across institutions.

Long-term neurocognitive outcomes are also underreported. Most studies use global functional measures such as the Glasgow Outcome Scale or return to school. Although these endpoints are clinically meaningful, they may fail to capture executive dysfunction, behavioral changes, learning difficulties, psychosocial challenges, and family burden. Pediatric patients who survive severe TBI may experience evolving deficits as developmental demands increase. Future studies should include formal neuropsychological testing, school performance, rehabilitation needs, quality of life, and caregiver-reported outcomes.

Overall, the existing literature supports a descriptive conclusion: bilateral, bifrontal, bitemporal, and cruciate decompressive craniectomy have been used in selected pediatric and young adult patients, can reduce ICP in some reports, and are associated with variable survival and functional recovery. However, current data do not establish comparative efficacy, optimal timing, or superiority of one bilateral decompressive technique.

### Limitations of evidence

There are significant limitations of this study, most obviously the small sample size of both number of studies and number of patients. In situations where treatment must be tailored specifically to each patient in their unique pathology, large data sets are often required to make strong conclusions on the population level. It is likely that even with large datasets, the management of these patients would still be largely individualized; however, larger data sets would allow a general standard of care to be more definitively reached. In addition to the small sample size of reported patients, the sample size of total studies that investigate these treatments for this pathology is low. This is likely due to both the rarity of these procedures, and the density of cases within a few pediatric hospitals. Compared with adult decompressive hemicraniectomies, these procedures on pediatric patients are typically only done at level 1 pediatric hospitals, which are much fewer in number than facilities that would offer these procedures for adults. Another limitation is the retrospective nature of these studies. In any research study investigating traumatic pathology, or critical care patients, prospective randomized controlled trials are challenging to facilitate. However, these prospective studies would allow stronger conclusions to be reached. A major limitation of this review is clinical and methodological heterogeneity across the included studies. Surgical indications, ICP thresholds, timing of decompression, operative technique, postoperative management, and outcome definitions varied substantially. These differences limit the ability to pool results or draw firm conclusions regarding the efficacy, timing, or superiority of any specific bilateral decompressive approach. Lastly many of these studies combined unilateral decompressive hemicraniectomy with bilateral approaches in pediatric patients. This combination of heterogeneous approaches makes it challenging to reach strong conclusions on the impact of bilateral approaches for this pathology. Also, the decision to exclude individual case studies to avoid anecdotal, highly selected cases driving quantitative impressions may have resulted in a smaller total sample population. Additional limitations include non-standardized outcome measurements and definitions and a large range of follow-up durations, from hospital discharge to several years post-operatively. Lastly, most data in the indexed studies were drawn from single centers, which increases the risk for bias and makes generalizability of study results more challenging. Large, multi-center, prospective studies are necessary to reach stronger conclusions with regards to malignant intracranial hypertension and the role for bi- and unilateral decompressive hemicraniectomy in pediatric patients. Further studies are necessary to clarify the nuances of DHC in pediatric intracranial hypertension. While current evidence confirms its important role in the management of medical management-resistant elevated ICP, extensive heterogeneity in primary interventions, timing of procedures, and procedural methods makes true comparisons difficult. Further retrospective studies may be useful in improving understanding of some of these features, however large, prospective multicenter trials will be the optimal way to investigate the utility of this procedure.

## Conclusions

Bilateral, bifrontal, bitemporal, and cruciate decompressive craniectomy have been reported as feasible salvage strategies for selected pediatric and young adult patients with refractory intracranial hypertension, most commonly after severe TBI. However, the current evidence base is small, heterogeneous, and frequently not technique-specific. As a result, this review cannot establish comparative efficacy, optimal timing, or superiority of bilateral decompression over other decompressive or medical strategies.

Existing studies suggest that postoperative ICP reduction is achievable and that some survivors attain meaningful functional recovery, but outcomes vary and complications such as hydrocephalus, subgaleal collections, infection, and need for additional surgery require careful surveillance. Larger prospective multicenter studies using standardized age criteria, ICP thresholds, operative classifications, complication definitions, and long-term neurocognitive outcomes are necessary.

## Supplementary Information

Below is the link to the electronic supplementary material.

Supplementary file1 (DOCX 8 KB)

## Data Availability

This is a systematic review of the literature. The extracted data is available in the manuscript tables. Other extracted data is available upon reasonable request to the corresponding author.

## References

[1] Zusman BE, Kochanek PM, Jha RM (2020) Cerebral edema in traumatic brain injury: a historical framework for current therapy. Curr Treat Options Neurol 22(3):9. 10.1007/s11940-020-0614-x 34177248 10.1007/s11940-020-0614-x PMC8223756

[2] Lolli V, Pezzullo M, Delpierre I, Sadeghi N (2016) MDCT imaging of traumatic brain injury. Br J Radiol 89(1061):20150849. 10.1259/bjr.20150849 26607650 10.1259/bjr.20150849 PMC4985461

[3] Ho ML, Rojas R, Eisenberg RL (2012) Cerebral edema. AJR Am J Roentgenol 199(3):W258–W273. 10.2214/AJR.11.8081 22915416 10.2214/AJR.11.8081

[4] Nakagawa K, Smith WS (2011) Evaluation and management of increased intracranial pressure. Continuum 17(5 Neurologic Consultation in the Hospital):1077–1093. 10.1212/01.CON.0000407061.25284.28 22809983 10.1212/01.CON.0000407061.25284.28

[5] Araki T, Yokota H, Morita A (2017) Pediatric traumatic brain injury: characteristic features, diagnosis, and management. Neurol Med Chir (Tokyo) 57(2):82–93. 10.2176/nmc.ra.2016-0191 28111406 10.2176/nmc.ra.2016-0191 PMC5341344

[6] van Loenhoud AC, Groot C, Vogel JW, van der Flier WM, Ossenkoppele R (2018) Is intracranial volume a suitable proxy for brain reserve? Alzheimers Res Ther 10(1):91. 10.1186/s13195-018-0408-5 30205838 10.1186/s13195-018-0408-5 PMC6134772

[7] Karibe H, Hayashi T, Narisawa A, Kameyama M, Nakagawa A, Tominaga T (2017) Clinical characteristics and outcome in elderly patients with traumatic brain injury: for establishment of management strategy. Neurol Med Chir (Tokyo) 57(8):418–425. 10.2176/nmc.st.2017-0058 28679968 10.2176/nmc.st.2017-0058 PMC5566701

[8] Carney N, Totten AM, O’Reilly C et al (2017) Guidelines for the management of severe traumatic brain injury, fourth edition. Neurosurgery 80(1):6–15. 10.1227/NEU.0000000000001432 27654000 10.1227/NEU.0000000000001432

[9] Gu J, Huang H, Huang Y, Sun H, Xu H (2019) Hypertonic saline or mannitol for treating elevated intracranial pressure in traumatic brain injury: a meta-analysis of randomized controlled trials. Neurosurg Rev 42(2):499–509. 10.1007/s10143-018-0991-8 29905883 10.1007/s10143-018-0991-8

[10] Hutchinson PJ, Kolias AG, Timofeev IS et al (2016) Trial of decompressive craniectomy for traumatic intracranial hypertension. N Engl J Med 375(12):1119–1130. 10.1056/NEJMoa1605215 27602507 10.1056/NEJMoa1605215

[11] Cooper DJ, Rosenfeld JV, Murray L et al (2011) Decompressive craniectomy in diffuse traumatic brain injury. N Engl J Med 364(16):1493–1502. 10.1056/NEJMoa1102077 21434843 10.1056/NEJMoa1102077

[12] Kan P, Amini A, Hansen K et al (2006) Outcomes after decompressive craniectomy for severe traumatic brain injury in children. J Neurosurg 105(5 Suppl):337–342. 10.3171/ped.2006.105.5.337 17328254 10.3171/ped.2006.105.5.337

[13] Manfiotto M, Beccaria K, Rolland A et al (2019) Decompressive craniectomy in children with severe traumatic brain injury: a multicenter retrospective study and literature review. World Neurosurg 129:e56–e62. 10.1016/j.wneu.2019.04.215 31054345 10.1016/j.wneu.2019.04.215

[14] Anderson V, Spencer-Smith M, Wood A (2011) Do children really recover better? Neurobehavioural plasticity after early brain insult. Brain 134(Pt 8):2197–2221. 10.1093/brain/awr103 21784775 10.1093/brain/awr103

[15] Taylor A, Butt W, Rosenfeld J et al (2001) A randomized trial of very early decompressive craniectomy in children with traumatic brain injury and sustained intracranial hypertension. Childs Nerv Syst 17(3):154–162. 10.1007/s003810000410 11305769 10.1007/s003810000410

[16] McHugh DC, Fiore SM, Strong N, Egnor MR (2019) Bifrontal biparietal cruciate decompressive craniectomy in pediatric traumatic brain injury. Pediatr Neurosurg 54(1):6–11. 10.1159/000495067 30605902 10.1159/000495067

[17] Ammar R, Chelly H, Kolsi F et al (2022) Decompressive craniectomy after traumatic brain injury: an observational study of 147 patients admitted in a Tunisian ICU. Interdiscip Neurosurg. 10.1016/j.inat.2021.101421

[18] Timofeev I, Kirkpatrick PJ, Corteen E et al (2006) Decompressive craniectomy in traumatic brain injury: outcome following protocol-driven therapy. Acta Neurochir Suppl 96:11–16. 10.1007/3-211-30714-1_3 16671414 10.1007/3-211-30714-1_3

[19] Korhonen TK, Suo-Palosaari M, Serlo W, Lahtinen MJ, Tetri S, Salokorpi N (2022) Favourable long-term recovery after decompressive craniectomy: the Northern Finland experience with a predominantly adolescent patient cohort. Childs Nerv Syst 38(9):1763–1772. 10.1007/s00381-022-05568-7 35739289 10.1007/s00381-022-05568-7 PMC9463249

[20] Konar SK, Dinesh YS, Shukla D et al (2024) Decompressive craniectomy in children: indications and outcome from a tertiary centre. Childs Nerv Syst 40(11):3757–3764. 10.1007/s00381-024-06513-6 38953913 10.1007/s00381-024-06513-6

[21] Grille P, Tommasino N (2015) Decompressive craniectomy in severe traumatic brain injury: prognostic factors and complications. Rev Bras Ter Intensiva 27(2):113–118. 10.5935/0103-507X.20150021 26340150 10.5935/0103-507X.20150021 PMC4489778

[22] Hubertus V, Finger T, Drust R et al (2022) Severe traumatic brain injury in children-paradigm of decompressive craniectomy compared to a historic cohort. Acta Neurochir (Wien) 164(5):1421–1434. 10.1007/s00701-022-05171-4 35305153 10.1007/s00701-022-05171-4 PMC9061678

[23] Rubiano AM, Villarreal W, Hakim EJ et al (2009) Early decompressive craniectomy for neurotrauma: an institutional experience. Ulus Travma Acil Cerrahi Derg 15(1):28–38 19130336 PMC3413286

[24] Sarma P, Shukla DP, Devi BI (2015) Bifrontal contusions: what is the best surgical treatment? Indian J Neurotrauma 12(2):103–106. 10.1055/s-0035-1570095

