# Supplementary material for: Bilateral decompressive craniectomy in pediatric patients: A systematic review
Source: Neurosurg Rev. 2026 May 30;49(1):426. doi: 10.1007/s10143-026-04335-5 (PMC13222325; doi:10.1007/s10143-026-04335-5)
Supplement: Supplementary file 1 — Supplementary file1 (DOCX 8 KB) [file 10143_2026_4335_MOESM1_ESM.docx]

Supplemental Table 1. Search Terms

| **Database** | Full Search Syntax | Number of Search Results |
| --- | --- | --- |
| PubMed | ("Decompressive Craniectomy"[MeSH Terms] OR "decompressive craniectomy"[Title/Abstract] OR "bifrontal craniectomy"[Title/Abstract] OR "bilateral craniectomy"[Title/Abstract] OR "craniectomy"[Title/Abstract]) AND ("Brain Injuries, Traumatic"[MeSH Terms] OR "traumatic brain injury"[Title/Abstract] OR TBI[Title/Abstract] OR "head injury"[Title/Abstract] OR "severe head trauma"[Title/Abstract] OR "intracranial hypertension"[MeSH Terms] OR "intracranial hypertension"[Title/Abstract] OR "raised intracranial pressure"[Title/Abstract] OR ICP[Title/Abstract]) AND ("Treatment Outcome"[MeSH Terms] OR outcome*[Title/Abstract] OR prognosis*[Title/Abstract] OR survival[Title/Abstract] OR "long-term outcome"[Title/Abstract] OR "neuropsychological outcome"[Title/Abstract] OR "cerebral oxygenation"[Title/Abstract] OR "predictive factor*"[Title/Abstract]) AND ("child"[MeSH Terms] OR "adolescent"[MeSH Terms] OR pediatric*[Title/Abstract] OR child*[Title/Abstract] OR adolescent*[Title/Abstract] OR youth[Title/Abstract]) | 75 |
| Embase | (decompressive AND 'craniectomy'/exp OR 'decompressive craniectomy':ti,ab) AND (bifrontal:ti,ab OR frontal:ti,ab OR frontotemporal:ti,ab OR bitemporal:ti,ab) AND ('child'/exp OR 'adolescent'/exp OR pediatric*:ti,ab OR paediatric*:ti,ab) AND ('outcome'/exp OR 'mortality'/exp OR 'prognosis'/exp OR 'risk factor':ti,ab OR 'predictive factor':ti,ab OR 'functional recovery':ti,ab) | 20 |
| Scopus | decompressive craniectomy AND pediatric OR child OR adolescent OR infant OR neonate AND bifrontal OR bitemporal OR bilateral AND outcome or mortality or prognosis or survival or recovery or disability or complication or morbidity or risk factor or predictive factor | 45 |

Supplemental Table 2. Mixed-age or Predominantly Adult Studies that were Excluded from the Primary Analysis

| **Study** | **Original relevance** | **Reason excluded from primary analysis** |
| --- | --- | --- |
| Timofeev 2006 | Protocol-driven bilateral frontotemporoparietal decompression | Mixed-age/adult cohort; median age 28 years, range 9-67; pediatric/young adult outcomes not separately extractable |
| Ammar 2022 | Decompressive craniectomy after TBI | Largely adult cohort; mean age 38.9 years, range 15-80; pediatric/young adult outcomes not separately extractable |
| Grille 2015 | Severe TBI decompressive craniectomy cohort | Mixed-age/adult cohort; mean age 31 ± 14 years; pediatric/young adult outcomes not separately extractable |
| Sarma 2015 | Bifrontal contusion operative management | Predominantly adult cohort; mean age 45 years, range 7-81; pediatric outcomes not separately extractable |
